# The Association of Thyrotropin and Autoimmune Thyroid Disease in Developing Papillary Thyroid Cancer

**DOI:** 10.1155/2017/5940367

**Published:** 2017-08-29

**Authors:** I-Shuan Lee, An-Tsz Hsieh, Ting-Wei Lee, Ting-I Lee, Yu-Mei Chien

**Affiliations:** ^1^Division of Endocrinology and Metabolism, Department of Internal Medicine, Wan Fang Hospital, Taipei Medical University, Taipei, Taiwan; ^2^Department of Internal Medicine, School of Medicine, College of Medicine, Taipei Medical University, Taipei, Taiwan; ^3^Division of Endocrinology and Metabolism, Department of Internal Medicine, Shuang Ho Hospital, Taipei Medical University, Taipei, Taiwan; ^4^Graduate Institute of Clinical Medicine, College of Medicine, Taipei Medical University, Taipei, Taiwan; ^5^Department of General Medicine, School of Medicine, College of Medicine, Taipei Medical University, Taipei, Taiwan

## Abstract

**Background:**

Papillary thyroid carcinoma (PTC) is the most common type of malignant thyroid neoplasm. However, the incidence of PTC with autoimmune thyroid disease (AITD) varies between studies. This study aims to investigate whether patients with AITD have increased incidence of PTC. We also analyzed the relationship of serum thyroid-stimulating hormone (TSH) levels and PTC in relation to AITD based on histopathological data.

**Methods:**

A total of 533 participants who underwent thyroidectomy were enrolled in this retrospective study based on clinicohistopathological data and known thyroid autoantibodies. Patients were divided into PTC and benign groups according to histopathologic diagnosis. Age, gender, body mass index, and serum TSH level before thyroidectomy were recorded.

**Results:**

Of the 533 enrolled patients, 159 (29.8%) were diagnosed with PTC, of which 38 (35.5%) had Hashimoto's thyroiditis (HT). More patients with HT were female, and patients with HT, Graves' disease, and thyroid nodules with higher TSH level had a higher incidence of PTC.

**Conclusions:**

A high proportion of the patients with PTC had HT. There was a trend that a higher serum TSH level was associated with a greater risk of thyroid cancer.

## 1. Introduction

Papillary thyroid carcinoma (PTC) is the most common malignant neoplasm of the thyroid gland. An association between thyroid autoimmunity and PTC has been reported in many previous studies [1, 2]. However, several cytological studies have not supported a link between thyroid nodules with autoimmune thyroid disease (AITD) and thyroid cancer [3, 4]. These inconsistent findings are probably due to selection bias since about 60% of patients with AITD undergo surgical interventions due to a suspicious cytology [3].

The association between PTC and HT was first described in 1955 by Dailey et al. [5, 6], and the concept of chronic inflammation as a risk factor for the development of malignancies has been well established [7, 8]. However, the effect of AITD on PTC remains controversial [9, 10]. More recently, some researchers have suggested that higher serum thyroid-stimulating hormone (TSH) levels, even within the normal range, are associated with a subsequent diagnosis of thyroid cancer in patients presenting with thyroid nodules [3]. This suggests that TSH may play a vital role in the development or progression of thyroid carcinomas. However, whether TSH levels are associated with the risk of PTC has yet to be elucidated.

Thus, the aim of this study was to investigate the prevalence of AITD in patients who underwent thyroidectomy and compare the clinicohistopathological characteristics of the patients with PTC with or without AITD. We further analyzed the association between TSH level and the risk of PTC based on the histopathological data.

## 2. Methods

This investigation was approved by the Research Ethics Committee of Wan Fang Hospital and Taipei Medical University (TMU JIRB Project no. N201603025).

### 2.1. Patients

**Figure 1:**
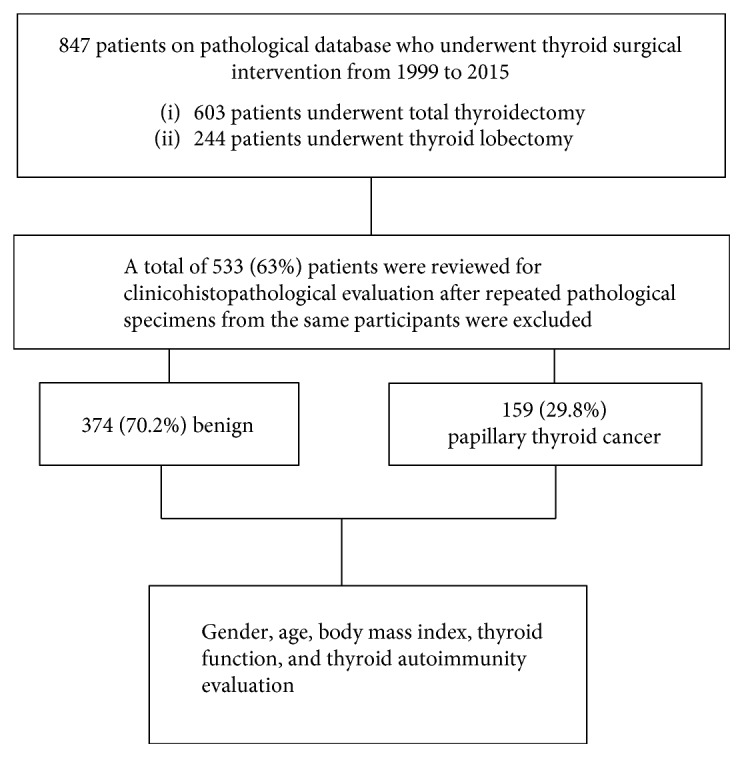
Schematic diagram of the study sample showing how the samples were collected and selected for analysis.

A total of 847 patients above 18 years of age and who underwent thyroid surgical intervention from January 1999 to December 2015 were identified in our pathological database. Of these patients, 603 underwent total thyroidectomy and 244 underwent thyroid lobectomy (Figure 1).

The histopathological records of 533 (63%) patients were retrospectively reviewed. Relevant cases had cytological evaluation of thyroid nodules by fine needle aspiration cytology (FNAC).

Patients with follicular adenoma (37 cases), follicular thyroid carcinoma (8 cases), poorly differentiated thyroid carcinoma (1 case), medullary thyroid cancer (1 case), and other tumors such as chondrosarcoma, schwannoma, and squamous cell carcinoma (3 cases) were excluded from the analysis.

### 2.2. Biochemical Assays

The following parameters were analyzed using Microsoft Excel® 2010 (Microsoft Corporation, Redmond, WA, United States): gender; age; body mass index (BMI); and thyroid function including serum TSH, free thyroxine (FT_4_), triiodothyronine (T_3_), thyroxine (T_4_), thyroid autoimmune antibodies including thyroid peroxidase antibody (anti-TPO Ab), antithyroglobulin antibody (anti-Tg Ab), TSH receptor antibody (TRAb), concomitant HT or concomitant Graves' disease, detailed histopathological data of papillary microcarcinoma (microPTC), TNM stage, association with lymph node metastasis, and concomitant other tumors or carcinoma.

### 2.3. Possible Predictors of Malignancy in Thyroid Nodules

#### 2.3.1. TSH

The serum TSH levels were divided into four groups by quartile within 90% confidence intervals of the reference limits in non-Gaussian distribution (5th percentile: 0.017 mIU/L, 25th percentile: 0.14 mIU/L, 50th percentile: 1.04 mIU/L, 75th percentile: 1.92 mIU/L, and 95th percentile: 4.52 mIU/L).

#### 2.3.2. Thyroid Autoimmunity

We recorded data on anti-TPO Ab, anti-Tg Ab, and TRAb for further subgroup analysis. The normal reference ranges for anti-TPO Ab and anti-Tg Ab levels were 0–34 IU/ml and 0–115 IU/ml, respectively. The normal reference range for TRAb was 0–15%.

#### 2.3.3. Indication of Surgical Intervention

All of the enrolled patients underwent thyroidectomy with one of the following indications: (1) FNAC results that were positive for malignancy; (2) FNAC results that were highly suspicious for malignancy; (3) nodular goiter with compression signs; (4) multiple nodular goiters with a progressive increase in size for elective thyroidectomy; and (5) patients with Graves' disease who selected surgical treatment.

### 2.4. Definitions and Pathology

HT was diagnosed as either positive serum thyroid autoantibodies (anti-TPO Ab or anti-Tg Ab) or by histopathological findings including the presence of diffuse lymphocyte infiltration, lymphoid follicles and germinal center formation, and Hürthle cell changes. Graves' disease was defined as a type of autoimmune problem that causes the thyroid gland to overproduce thyroid hormones by thyroid-stimulating immunoglobulins. MicroPTC was defined as a tumor with a diameter ≤ 1.0 cm in histological examinations. Clinicopathological staging was performed according to the American Joint Committee on Cancer TNM staging system (7th Edition; New York, NY: Springer-Verlag; 2010). Lymph node status was assessed according to the pathological evidence of metastasis in the lymph nodes removed during the operation.

Synchronous malignancy was defined as the diagnosis of a nonthyroidal malignancy or tumor within 6 months of diagnosis of PTC. Synchronous nonthyroidal malignancies or tumors were also assessed in the enrolled patients.

### 2.5. Statistical Analysis

Descriptive analysis was used to summarize the data. Continuous variables with normal distribution were expressed as means and standard deviations, while categorical variables were expressed as frequencies and percentages. Multivariate analysis of variance was analyzed by one-way ANOVA with Scheffé post hoc tests. Pearson's correlation coefficient was used to evaluate the relationships between positive TRAb and stages as well as the subtypes of the PTC patients. All statistical analyses were performed using SPSS software version 22.0 (SPSS Inc., Chicago, IL, USA). A *p* value of <0.05 was considered to be statistically significant.

## 3. Results

### 3.1. Clinicohistopathological Characteristics according to the Pathological Results

As shown in Figure 1, a total of 533 patients were included in this study, 159 of whom had PTC and 374 had benign lesions. Significantly more female patients had PTC than benign lesions (88.7% versus 80%, *p* = 0.02).

Analysis of the clinicohistopathological characteristics according to the pathological results of all patients who underwent thyroidectomy (Table 1) showed that the benign group had significantly higher values FT_4_ (1.8 ng/dl versus 1.3 ng/dl, *p* = 0.003), T_3_ (144 ng/dl versus 111 ng/dl, *p* = 0.007), and positive TRAb (84% versus 9%, *p* = 0.001) compared to the PTC group. Based on our pathological database, the results in the benign group were derived from the patients with Graves' disease who received surgery for treatment. However, the serum TSH level was similar between the two groups (1.6 mIU/L versus 1.9 mIU/L, *p* = 0.702). 12.6% of the patients with PTC had synchronous nonthyroid malignancies or tumors (Table 1) compared to the benign group.

As shown in Table 2, the commonly associated synchronous nonthyroid malignancies or tumors in our study were breast ductal carcinoma (30%), colon hyperplastic polyp (15%), colon tubular adenoma (10%), colon tubulovillous adenoma (10%), and colon adenocarcinoma (10%).

### 3.2. Clinicohistopathological Results of the PTC Group

A total of 127 patients with PTC were compared with 241 patients with benign lesions (Table 3). Both the PTC and benign groups had a similar female predominance (88.2% versus 82.2%, resp.). The mean ages of the patients in the PTC and benign groups were 47.7 ± 12.7 and 46.1 ± 14.7 years, respectively. The patients with PTC had a significantly higher serum TSH level (1.59 mIU/L versus 0.96 mIU/L, *p* = 0.001) and lower FT_4_ (1.3 ng/dl versus 1.8 ng/dl, *p* = 0.006) and T_3_ (112 ng/dl versus 144 ng/dl, *p* = 0.01) levels compared with those in the benign group (Table 3). There were no significant differences in anti-TPO Ab or anti-Tg Ab level between the two groups but a significantly higher level of TRAb (69.1% versus 29.6%, *p* = 0.001) in the benign group (Table 3). Of the patients with PTC, 12.6% had synchronous nonthyroidal malignancies or tumors.

The prevalence of PTC was predicted by subgrouping according to serum TSH level. The results of subgroup analysis of TSH levels showed a higher prevalence of PTC in the subgroup with a higher TSH level.

### 3.3. Related Variables in Patients with PTC Excluding Those with Graves' Disease Receiving Thyroidectomy

As shown in Table 4, a total of 144 patients with PTC after Graves' disease who underwent thyroidectomy were excluded and they were compared with 263 patients with benign lesions. Both the PTC and benign groups had a similar female predominance (87.5% versus 84%, resp.). The mean ages of the patients in the PTC and benign groups were 47.2 ± 12.9 and 49.2 ± 14.5 years, respectively. There were no significant differences in anti-TPO Ab, anti-Tg Ab, or TRAb levels between the two groups. Of the patients with PTC excluding those with Graves' disease who underwent thyroidectomy, 13.2% had synchronous nonthyroidal malignancies or tumors. Moreover, the subanalysis of TSH levels showed a significantly higher risk of PTC when the TSH values were higher.

### 3.4. PTC Patients with Coexisting HT

We then performed subgroup analysis of the patients with PTC with coexisting HT (Table 5). A total of 38 patients with PTC had coexisting HT, and we compared them with 70 patients with benign lesions. The results showed that the patients with PTC had a higher female predominance than the benign group (94.7% versus 80%, *p* = 0.04). The mean ages of the PTC and benign groups were 44.2 ± 10.5 and 40.3 ± 16 years, respectively. The benign group had higher FT_4_ and T_3_ levels compared with the PTC group (2.4 ng/dl versus 1.3 ng/dl, *p* = 0.006 and 191 ng/dl versus 126 ng/dl, *p* = 0.02, resp.). The PTC group also had a lower rate of positive TRAb (82.0% versus 27.3%, *p* = <0.001), but this was not related to lymph node metastasis. Of the patients with PTC with coexisting HT, 15.8% had synchronous nonthyroidal malignancies or tumors.

In subgroup analysis by TSH level, the subgroup with higher TSH levels had a higher risk of PTC. The prevalence of PTC with coexisting HT was predicted by subgrouping according to serum TSH level.

### 3.5. Subgroup Analysis of All Patients with Positive TRAb

We further performed subgroup analysis according to only those with positive TRAb. A total of nine patients had PTC, and 84 had benign lesions. The female predominance of the PTC and benign groups was 100% versus 70.2%, respectively (*p* = 0.057). The mean age of the patients with PTC was significantly older than that of the benign group (48.89 ± 9.6 versus 36.69 ± 12.4 years, *p* = 0.006). The PTC group also had a higher proportion of microPTC (55.6%) (Table 6). Of the patients with PTC with positive TRAb, 11.1% had synchronous nonthyroidal malignancies or tumors. In addition, in the subgroup analysis according to TSH level, the subgroup with higher TSH levels had a higher risk of PTC.

### 3.6. Correlation between Different Stages and Subtypes of PTC with Positive TRAb

Among the PTC patients with positive TRAb, eight patients were classic type of PTC and one patient had a follicular variant type. All of these nine PTC patients with positive TRAb were in stage 1 TNM classification (Table 6). Our clinical study showed that PTC patients with positive TRAb were not positively correlated with the stages of PTC (*r* = −0.322, *p* = 0.078). In addition, there was no evidence of a more aggressive phenotype of PTC in patients with Graves' disease.

## 4. Discussion

This study explored the significant associations of PTC in patients with AITD. Approximately, 29.8% of the patients with AITD in this present study were affected by PTC. However, higher values of FT_4_, T_3_, and positive TRAb were found in patients with benign lesions than in patients with PTC. Some investigations have suggested that thyroid cancer is more aggressive in Graves' disease [11], but this is not a ubiquitous phenomenon [12]. Moreover, it is not yet known whether Graves' disease with positive TRAb is correlated with a more aggressive phenotype of differentiated thyroid cancer. Thus, we further analyzed all PTC patients with positive and negative TRAb results. We found in our subgroup analysis that these PTC patients with positive TRAb were diagnosed at an earlier clinicopathological stage (stage 1), and we found no correlation between the aggressive phenotype of differentiated thyroid cancer and Graves' disease. The inconsistencies between the results might probably be due to some confounding factors such as the environment, genetics, or other unknown factors [13].

The prevalence of thyroid cancer in patients with HT also varies, ranging from 4.8% to 60% [1, 9, 14]. The presence of HT causes large variations in the ultrasonographic appearance of the thyroid glands that make it more difficult to differentiate between benign and malignant nodules [15]. In our cases, when we excluded patients who received thyroidectomy for the treatment of Graves' disease from our analysis, we found that histopathological characteristics suggestive of HT were significantly associated with PTC. In addition, we found that a higher level of serum TSH was associated with a greater risk of thyroid cancer. However, we found no strong evidence of an increased risk of multifocal and capsular invasions in HT as has been previously reported in other studies [16, 17]. Similarly, we found that HT was not related to a higher risk of lymph node metastasis or advanced cancer staging. Whether HT has an immune response that may act as a protective mechanism for the aggressiveness of PTC remains unclear.

In the subgroup analysis of patients with HT, defined as either positive thyroid autoantibodies (anti-TPO and Tg Ab) or histopathological characteristics suggestive of HT, the patients with positive TRAb and higher values of FT_4_ and T_3_ tended to have a more benign pathological diagnosis. We also found that the patients with HT with higher levels of serum TSH had a higher incidence of PTC. Serum TSH has been reported to act as a promoter of malignancy in AITD [18–20]. Due to the coexistence of anti-TPO Ab and TRAb in AITD, our results imply that clinicians should be cautious when evaluating thyroid nodules in patient with positive TRAb and a relatively high serum TSH level.

As shown in the previous literature, most thyroid cancers which are associated with hyperthyroidism are usually microcarcinomas [21]. In our subgroup analysis of patients with positive TRAb, we found nine cases with PTC who were diagnosed at stage 1. The higher incidence of microPTC (55.6%) in our cases may also be related to the early detection of PTC in Graves' disease. Clinicians should therefore consider screening selected patients with Graves' disease for thyroid nodules, but should also be aware of potentially overdiagnosing microPTC [22].

Several studies have demonstrated an approximated 11% to 30% risk of developing various cancers such as breast, prostate, kidney, salivary, scrotal, neural, and leukemia after primary thyroid cancer [23, 24]. A prevalence rate of approximately 14% of synchronous or antecedent nonthyroidal cancers has also been reported in surgically treated PTC patients [25]. We found a 12.6% incidence rate of synchronous nonthyroidal malignancies or tumors in our PTC patients, compared to 15.8% in our PTC patients with coexisting HT. Most of the associated synchronous tumors in our study were breast ductal carcinoma and colon tubulovillous adenoma or carcinoma. These findings suggest that there is a risk of developing synchronous nonthyroidal malignancies or tumors after surgery for PTC. Surgeons should be aware of the close association between synchronous nonthyroidal malignancies or tumors and PTC, and further evaluations after PTC surgery should be performed.

### 4.1. Study Limitations

There are several limitations in this study. First, this is a retrospective chart review from a single center, and the sample sizes of our subgroups were relatively small. Second, there is no complete information about the medication of the patients, whether they are receiving LT_4_ treatment or antithyroid drugs at the time of measurement. Third, we were unable to obtain preoperative ultrasonographic characteristics of the nodules in patients with AITD to compare it with the clinicopathological results of our cases. Fourth, the identification of synchronous nonthyroidal malignancies or tumors was made according to the medical provided in the medical records, and the true prevalence may have been higher than the reported cases.

## 5. Conclusions

A high proportion of the cases with PTC also had HT. Results showed a trend in which a higher serum TSH level was associated with a higher risk of thyroid cancer.

## Figures and Tables

**Table 1:** Clinicohistopathological characteristics according to pathological results of patients enrolled in the study.

Result	Benign (*N* = 374)	PTC (*N* = 159)	*p* value
Gender, female (%)	300 (80.2)	141 (88.7)	0.018
Age (years)	45.33 ± 15.1	47.11 ± 12.8	0.194
BMI (kg/m^2^)	23.7 ± 3.7	24.2 ± 4.2	0.241
TSH (mIU/L)	1.6 ± 5.2	1.9 ± 1.4	0.702
FT_4_ (ng/dl)	1.8 ± 1.4	1.3 ± 0.7	0.003
T_4_ (*μ*g/dl)	9.0 ± 8.8	7.8 ± 2.1	0.431
T_3_ (ng/dl)	144.0 ± 82.5	111.4 ± 29.6	0.007
Anti-TPO Ab positive (%)	66 (38.2)	27 (29.7)	0.172
Anti-Tg Ab positive (%)	10 (16.4)	14 (20.0)	0.598
TRAb positive (%)	84 (73.7)	9 (29.0)	0.000
MicroPTC (%)		34 (21.4)	
Stage	1	115 (72.3)	
	2	11 (6.9)	
	3	12 (7.5)	
	4	21 (13.2)	
LN metastasis (%)		47 (29.6)	
Synchronous nonthyroidal malignancies or tumors (%)		20 (12.6)	

PTC: papillary thyroid cancer; BMI: body mass index; TSH: thyroid-stimulating hormone; FT_4_: free thyroxine; T_4_: thyroxine; T_3_: triiodothyronine; anti-TPO Ab: thyroid peroxidase antibodies; anti-TG Ab: antithyroglobulin antibody; TRAb: TSH receptor antibody; HT: Hashimoto's thyroiditis; microPTC: papillary thyroid microcarcinoma; LN: lymph node.

**Table 2:** List of synchronous nonthyroid malignancies or tumors.

Synchronous nonthyroid malignancies or tumors	Cases (%)
Breast ductal carcinoma	6 (30)
Colon hyperplastic polyp	3 (15)
Colon tubular adenoma	2 (10)
Colon tubulovillous adenoma	2 (10)
Colon adenocarcinoma	2 (10)
Lung adenocarcinoma	1 (5)
Endometrial carcinoma	1 (5)
Cervical carcinoma	1 (5)
Adrenal cortical adenoma	1 (5)
Parotid pleomorphic adenoma	1 (5)

**Table 3:** Clinicohistopathological results of papillary thyroid cancer classified according to serum TSH level.

Result	Benign (*N* = 241)	PTC (*N* = 127)	*p* value
Gender, female (%)	198 (82.2)	112 (88.2)	0.132
Age (years)	46.1 ± 14.7	47.7 ± 12.7	0.280
BMI (kg/m^2^)	23.7 ± 3.6	24.0 ± 4.0	0.561
TSH (mIU/L)	0.96 ± 0.99	1.59 ± 0.97	0.000
TSH with group			0.000
(i) Group 1 (0.017–0.14)	77 (32.0)	7 (5.5)	
(ii) Group 2 (0.14–1.04)	70 (29.0)	31 (24.4)	
(iii) Group 3 (1.04–1.92)	53 (22.0)	49 (38.4)	
(iv) Group 4 (1.92–4.52)	41 (17.0)	40 (31.5)	
FT_4_ (ng/dl)	1.8 ± 1.4	1.3 ± 0.7	0.006
T_4_ (*μ*g/dl)	9.2 ± 9.2	7.9 ± 2.1	0.398
T_3_ (ng/dl)	144.3 ± 84.4	111.9 ± 30.1	0.010
Anti-TPO Ab positive (%)	52 (34.7)	26 (31.3)	0.607
Anti-Tg Ab positive (%)	8 (14.8)	11 (17.7)	0.674
TRAb positive (%)	65 (69.1)	8 (29.6)	0.000
MicroPTC (%)		30 (23.6)	
Stage	1	94 (74.0)	
	2	7 (23.6)	
	3	10 (7.9)	
	4	16 (12.6)	
LN metastasis (%)		37 (29.1)	
Synchronous nonthyroidal malignancies or tumors (%)		16 (12.6)	

PTC: papillary thyroid cancer; BMI: body mass index; TSH: thyroid-stimulating hormone; FT_4_: free thyroxine; T_4_: thyroxine; T_3_: triiodothyronine; anti-TPO Ab: thyroid peroxidase antibodies; anti-TG Ab: antithyroglobulin antibody; TRAb: TSH receptor antibody; HT: Hashimoto's thyroiditis; microPTC: papillary thyroid microcarcinoma; LN: lymph node.

**Table 4:** Univariate analysis: related variables in patients with papillary thyroid cancer excluding those with Graves' disease who received thyroidectomy.

Result	Benign (*N* = 263)	PTC (*N* = 144)	*p* value
Gender, female (%)	221 (84.0)	126 (87.5)	0.346
Age (years)	49.2 ± 14.5	47.2 ± 12.9	0.165
BMI (kg/m^2^)	23.9 ± 3.6	24.1 ± 4.1	0.638
TSH (mIU/L)	1.9 ± 5.15	1.9 ± 1.4	0.975
TSH with group			0.003
(i) Group 1 (<0.14)	19 (10.6)	3 (2.5)	
(ii) Group 2 (0.14–1.04)	64 (35.8)	29 (24.2)	
(iii) Group 3 (1.04–1.92)	48 (26.8)	46 (38.3)	
(iv) Group 4 (>1.92)	48 (26.8)	42 (35.0)	
FT_4_ (ng/dl)	1.3 ± 0.52	1.2 ± 0.2	0.130
T_4_ (*μ*g/dl)	8.9 ± 10.6	7.4 ± 1.2	0.445
T_3_ (ng/dl)	106.9 ± 40.7	106.5 ± 21.9	0.955
Anti-TPO Ab positive (%)	18 (15.9)	22 (26.8)	0.063
Anti-Tg Ab positive (%)	4 (8.9)	11 (17.2)	0.219
TRAb positive (%)	7 (24.1)	2 (9.5)	0.192
MicroPTC (%)		26 (18.1)	
Stage	1	101 (70.1)	
	2	11 (7.6)	
	3	11 (7.6)	
	4	21 (14.6)	
LN metastasis (%)		46 (31.9)	
Synchronous nonthyroidal malignancies or tumors (%)		19 (13.2)	

PTC: papillary thyroid cancer; BMI: body mass index; TSH: thyroid-stimulating hormone; FT_4_: free thyroxine; T_4_: thyroxine; T_3_: triiodothyronine; anti-TPO Ab: thyroid peroxidase antibodies; anti-TG Ab: antithyroglobulin antibody; TRAb: TSH receptor antibody; HT: Hashimoto's thyroiditis; microPTC: papillary thyroid microcarcinoma; LN: lymph node.

**Table 5:** Subgroup analysis of patients with coexisting Hashimoto's thyroiditis.

Result	Benign (*N* = 70)	PTC (*N* = 38)	*p* value
Gender, female (%)	56 (80.0)	36 (94.7)	0.040
Age (years)	40.3 ± 16.0	44.2 ± 10.5	0.181
BMI (kg/m^2^)	23.8 ± 4.1	23.7 ± 4.4	0.917
TSH (mIU/L)	1.9 ± 8.3	1.8 ± 1.7	0.923
TSH with group			0.000
(i) Group 1 (<0.14)	41 (60.3)	3 (8.8)	
(ii) Group 2 (0.14–1.04)	10 (14.7)	6 (17.6)	
(iii) Group 3 (1.04–1.92)	5 (7.4)	13 (38.2)	
(iv) Group 4 (>1.92)	12 (17.6)	12 (35.3)	
FT_4_ (ng/dl)	2.4 ± 1.95	1.3 ± 0.5	0.006
T_4_ (*μ*g/dl)	9.2 ± 4.2	7.97 ± 1.3	0.362
T_3_ (ng/dl)	190.8 ± 102.2	125.9 ± 34.2	0.024
TRAb positive (%)	41 (82.0)	3 (27.3)	0.000
MicroPTC (%)		10 (26.3)	
Stage	1	31 (81.6)	
	2	2 (5.3)	
	3	2 (5.3)	
	4	3 (7.9)	
LN metastasis (%)		13 (34.2)	
Synchronous nonthyroidal malignancies or tumors (%)		6 (15.8)	

PTC: papillary thyroid cancer; BMI: body mass index; TSH: thyroid-stimulating hormone; FT_4_: free thyroxine; T_4_: thyroxine; T_3_: triiodothyronine; anti-TPO Ab: thyroid peroxidase antibodies; anti-TG Ab: antithyroglobulin antibody; TRAb: TSH receptor antibody; HT: Hashimoto's thyroiditis; microPTC: papillary thyroid microcarcinoma; LN: lymph node.

**Table 6:** Subgroup analysis of patients with positive TSH receptor antibody.

Result	Benign (*N* = 84)	PTC (*N* = 9)	*p* value
Gender, female (%)	59 (70.2)	9 (100)	0.057
Age (years)	36.7 ± 12.5	48.9 ± 9.6	0.006
BMI (kg/m^2^)	23.6 ± 4.3	24.0 ± 5.5	0.793
TSH (mIU/L)	0.9 ± 4.9	1.1 ± 1.3	0.915
TSH with group			0.018
(i) Group 1 (<0.14)	67 (81.7)	3 (37.5)	
(ii) Group 2 (0.14–1.04)	4 (4.9)	1 (12.5)	
(iii) Group 3 (1.04–1.92)	3 (3.7)	2 (25)	
(iv) Group 4 (>1.92)	8 (9.8)	2 (25)	
FT_4_ (ng/dl)	2.7 ± 2.1	1.7 ± 1.2	0.206
T_4_ (*μ*g/dl)	9.7 ± 5.0	10.7 ± 4.9	0.707
T_3_ (ng/dl)	194.9 ± 96.4	167.5 ± 50.6	0.578
Anti-TPO Ab positive (%)	41 (77.4)	3 (42.9)	0.052
Anti-Tg Ab positive (%)	6 (46.2)	1 (33.3)	0.687
MicroPTC (%)		5 (55.6)	
Stage	1	9 (100)	
	2	0 (0)	
	3	0 (0)	
	4	0 (0)	
LN metastasis (%)		1 (11.1)	
Synchronous nonthyroidal malignancies or tumors (%)		1 (11.1)	

PTC: papillary thyroid cancer; BMI: body mass index; TSH: thyroid-stimulating hormone; FT_4_: free thyroxine; T_4_: thyroxine; T_3_: triiodothyronine; anti-TPO Ab: thyroid peroxidase antibodies; anti-TG Ab: antithyroglobulin antibody; HT: Hashimoto's thyroiditis; microPTC: papillary thyroid microcarcinoma; LN: lymph node.

## References

[1] Paparodis R., Imam S., Todorova-Koteva K., Staii A., Jaume J. C. (2014). Hashimoto’s thyroiditis pathology and risk for thyroid cancer. *Thyroid*.

[2] Staniforth J. U., Erdirimanne S., Eslick G. D. (2016). Thyroid carcinoma in Graves’ disease: a meta-analysis. *International Journal of Surgery*.

[3] Maria Grazia Castagna V. B., Memmo S., Maino F. (2014). Nodules in autoimmune thyroiditis are associated with increased risk of thyroid cancer in surgical series but not in cytological series: evidence for selection bias. *The Journal of Clinical Endocrinology and Metabolism*.

[4] Sohn S. Y., Kim H. J., Jang H. W., Kim S. W., Chung J. H. (2014). Lack of association between high serum thyroid-stimulating hormone level and risk of papillary thyroid microcarcinomas. *Head & Neck*.

[5] Dailey M. E., Lindsay S., Skahen R. (1955). Relation of thyroid neoplasms to Hashimoto disease of the thyroid gland. *A.M.A. Archives of Surgery*.

[6] Azizi G., Keller J. M., Lewis M. (2014). Association of Hashimoto’s thyroiditis with thyroid cancer. *Endocrine Related Cancer*.

[7] Weber F. (2014). Lymphocytes and thyroid cancer: more to it than meets the eye?. *Endocrine Related Cancer*.

[8] Ehlers M., Schott M. (2014). Hashimoto’s thyroiditis and papillary thyroid cancer: are they immunologically linked?. *Trends in Endocrinology & Metabolism*.

[9] Lee J. H., Kim Y., Choi J. W., Kim Y. S. (2013). The association between papillary thyroid carcinoma and histologically proven Hashimoto’s thyroiditis: a meta-analysis. *European Journal of Endocrinology*.

[10] Jankovic B., Le K. T., Hershman J. M. (2013). Clinical review: Hashimoto’s thyroiditis and papillary thyroid carcinoma: is there a correlation?. *The Journal of Clinical Endocrinology and Metabolism*.

[11] Yano Y., Shibuya H., Kitagawa W., Nagahama M., Sugino K., Ito K. (2007). Recent outcome of Graves’ disease patients with papillary thyroid cancer. *European Journal of Endocrinology*.

[12] Ahn D., Sohn J. H., Jeon J. H., Park J. (2014). Preoperative subclinical hyperthyroidism in patients with papillary thyroid carcinoma. *Clinical and Experimental Otorhinolaryngology*.

[13] Pazaitou-Panayiotou K., Perros P., Boudina M. (2008). Mortality from thyroid cancer in patients with hyperthyroidism: the Theagenion Cancer Hospital experience. *European Journal of Endocrinology*.

[14] Girardi F. M., Barra M. B., Zettler C. G. (2015). Papillary thyroid carcinoma: does the association with Hashimoto’s thyroiditis affect the clinicopathological characteristics of the disease?. *Brazilian Journal of Otorhinolaryngology*.

[15] Kamile Gul A. D., Kiyak G., Ersoy P. E., Ugras N. S., Ersoy R., Cakir B. (2010). The association between thyroid carcinoma and Hashimoto’s thyroiditis: the ultrasonographic and histopathologic characteristics of malignant nodules. *Thyroid*.

[16] Singh B., Shaha A. R., Trivedi H., Carew J. F., Poluri A., Shah J. P. (1999). Coexistent Hashimoto’s thyroiditis with papillary thyroid carcinoma: impact on presentation, management, and outcome. *Surgery*.

[17] Zhu F., Shen Y. B., Li F. Q., Fang Y., Hu L., Wu Y. J. (2016). The effects of Hashimoto thyroiditis on lymph node metastases in unifocal and multifocal papillary thyroid carcinoma: a retrospective Chinese cohort study. *Medicine (Baltimore)*.

[18] Megan Rist Haymart D. J. R., Leverson G. E., Elson D. F., Sippel R. S., Jaume J. C., Chen H. (2008). Higher serum thyroid stimulating hormone level in thyroid nodule patients is associated with greater risks of differentiated thyroid cancer and advanced tumor stage. *The Journal of Clinical Endocrinology and Metabolism*.

[19] Boelaert K. (2009). The association between serum TSH concentration and thyroid cancer. *Endocrine Related Cancer*.

[20] Fiore E., Rago T., Provenzale M. A. (2009). Lower levels of TSH are associated with a lower risk of papillary thyroid cancer in patients with thyroid nodular disease: thyroid autonomy may play a protective role. *Endocrine Related Cancer*.

[21] Kikuchi S., Noguchi S., Yamashita H., Uchino S., Kawamoto H. (2006). Prognosis of small thyroid cancer in patients with Graves’ disease. *The British Journal of Surgery*.

[22] Wei S., Baloch Z. W., LiVolsi V. A. (2014). Thyroid carcinoma in patients with Graves’ disease: an institutional experience. *Endocrine Pathology*.

[23] Ronckers C. M., McCarron P., Ron E. (2005). Thyroid cancer and multiple primary tumors in the SEER cancer registries. *International Journal of Cancer*.

[24] Sandeep T. C., Strachan M. W., Reynolds R. M. (2006). Second primary cancers in thyroid cancer patients: a multinational record linkage study. *The Journal of Clinical Endocrinology and Metabolism*.

[25] Murray S., Schneider D. F., Bauer P. S., Sippel R. S., Chen H. (2013). Synchronous and antecedent non-thyroidal malignancies in patients with papillary thyroid carcinoma. *Journal of the American College of Surgeons*.

